# A new screening system for entry inhibitors based on cell-to-cell transmitted syncytia formation mediated by self-propagating hybrid VEEV-SARS-CoV-2 replicon

**DOI:** 10.1080/22221751.2022.2030198

**Published:** 2022-02-04

**Authors:** Na Li, Xiao-Ling Chen, Qi Li, Zhe-Rui Zhang, Cheng-Lin Deng, Bo Zhang, Xiao-Dan Li, Han-Qing Ye

**Affiliations:** a Key Laboratory of Special Pathogens and Biosafety, Wuhan Institute of Virology, Center for Biosafety Mega-Science, Chinese Academy of Sciences, Wuhan, People’s Republic of China; b University of Chinese Academy of Sciences, Beijing, People’s Republic of China; c College of Pharmacy and Drug Discovery Center for Infectious Diseases, Nankai University, Tianjin, People’s Republic of China; d Hunan Normal University, School of Medicine, Changsha, People’s Republic of China

**Keywords:** SARS-CoV-2, VEEV, syncytia, spike, inhibitors

## Abstract

The extremely high transmission rate of SARS-CoV-2 and severe cases of COVID-19 pose the two critical challenges in the battle against COVID-19. Increasing evidence has shown that the viral spike (S) protein-driven syncytia may be responsible for these two events. Intensive attention has thus been devoted to seeking S-guided syncytium inhibitors. However, the current screening campaigns mainly rely on either live virus-based or plasmid-based method, which are always greatly limited by the shortage of high-level biosafety BSL-3 facilities or too much labour-intensive work. Here, we constructed a new hybrid VEEV-SARS-CoV-2-S-eGFP reporter vector through replacement of the structural genes of Venezuelan equine encephalitis virus (VEEV) with the S protein of SARS-CoV-2 as the single structural protein. VEEV-SARS-CoV-2-S-eGFP can propagate steadily through cell-to-cell transmission pathway in S- and ACE2-dependent manner, forming GFP positive syncytia. In addition, a significant dose-dependent decay in GFP signals was observed in VEEV-SARS-CoV-2-S-eGFP replicating cells upon treatment with SARS-CoV-2 antiserum or entry inhibitors, providing further evidence that VEEV-SARS-CoV-2-S-eGFP system is highly sensitive to characterize the anti-syncytium-formation activity of antiviral agents. More importantly, the assay is able to be performed in a BSL-2 laboratory without manipulation of live SARS-CoV-2. Taken together, our work establishes a more convenient and efficient VEEV-SARS-CoV-2-S-eGFP replicating cells-based method for rapid screening of inhibitors blocking syncytium formation.

## Introduction

Since a novel coronavirus disease (COVID-19) caused by severe acute respiratory syndrome coronavirus type 2 (SARS-CoV-2) was first reported in late 2019 in Wuhan, China [1,2], it has spread worldwide rapidly and resulted in more than 280 million confirmed cases with over 5 million mortalities (https://www.worldometers.info/coronavirus/). Despite unprecedented research efforts against COVID-19 in the whole world, there are no efficacious therapeutics available for SARS-CoV-2 infection up to now.

The SARS-CoV-2 genome is a single-stranded, positive-sense RNA approximately 29.9 kb in length comprising two large polyproteins: ORF1a and ORF1ab; four structural proteins: spike (S) protein, membrane (M) protein, envelope (E) protein and nucleocapsid (N) protein; and eight accessory proteins: ORF3a, ORF3b, ORF6, ORF7a, ORF7b, ORF8a, ORF8b, and ORF9b [3,4]. Among them, the S protein is a viral glycoprotein protruding from the virion surface as a homotrimer. It contains two functional subunits S1 and S2 responsible for viral attachment and entry into target cells. The S1 subunit comprises the receptor-binding domain (RBD) that mediates the binding to the host angiotensin-converting enzyme 2 (ACE2) receptors, while the C-terminal S2 subunit plays a key role in virus-cell membrane fusion [5,6]. Besides mediating virus entry, excess S protein present at the plasma membrane can stimulate the fusion with neighbouring cells expressing ACE2 and form syncytia. It has been well-documented that such syncytium-inducing phenotypes are associated with clinically severe cases of COVID-19 as well as the extremely rapid transmission rate of SARS-CoV-2 [7,8]. Additionally, Asarnow *et al.* found that some neutralizing antibodies efficiently blocking the S protein-ACE2 interaction had the ability to enhance syncytium formation [9], and thus displaying much less viral neutralizing activity, which highlights the necessity to evaluate the efficacy of antivirals to inhibit cell–cell fusion especially in the treatment of severe COVID-19.

Currently, the most commonly used methods for screening antivirals targeting the events associated with cell–cell fusion and syncytium formation mainly include live virus-based and plasmid-based. The former relies on a specialized biosafety level-3 (BSL-3) containment facility to manipulate SARS-CoV-2 [10], and the whole process takes a relatively long time (2-4 days) to complete [11]. In addition, access to BSL-3 facility is not available or very limited for most researchers, which also impedes related investigations. The latter, though circumventing biosafety issues, is performed through repeated transfection of S-expressing-plasmid, which is tedious and costly. Therefore, it is important to develop a more convenient and efficient alternative for rapid screening of inhibitors blocking syncytium formation.

The alphaviruses from the *Togaviridae* family are positive-strand RNA viruses including Venezuelan equine encephalitis virus (VEEV), Sindbis virus (SINV) and Semliki Forest virus (SFV). Due to the self-amplification feature and the broad host range, the alphavirus replicon vectors are widely used for gene expression [12]. Through replacement of the alphavirus structural proteins with the gene of interest, high-level of antigens expression and the resulting specific cellular, humoral, and mucosal immune responses are induced, making alphavirus replicon vectors attractive for gene-based vaccines for many infectious diseases [13–15]. Among the alphavirus replicon vectors, VEEV has been shown to induce potent immune responses in several animal models [16–18]. In our previous study, a VEEV replicon derived, self-propagating replicon particles (PRPs) or infectious microvesicles (iMVs) expressing RABV-G protein, designated as VEEV-RABV-G, are proved to be safe and effective in mouse model, and can be a promising RABV vaccine candidate for human and domestic animal use [19].

In contrast to the previous study, a new VEEV self-replicating replicon expressing S glycoprotein of SARS-CoV-2 (VEEV-SARS-CoV-2-S-eGFP) could only form syncytium through cell-to-cell transmission without iMVs production. VEEV-SARS-CoV-2-S-eGFP could be used as a surrogate system for screening antivirals blocking S-dependent syncytium formation. Our results indicate that VEEV-SARS-CoV-2-S-eGFP was able to generate visible GFP-positive syncytia in S- and ACE2- dependent manner, with great similarity to the authentic SARS-CoV-2, but did not release free infectious iMVs even had undergone passages in ACE2-positive Vero-E6 cells for as high as 20 rounds. It was demonstrated this system can be exploited to identify the cell–cell fusion inhibitors and neutralization antibodies. More importantly, the assay was able to be performed in a BSL-2 laboratory without manipulation of live SARS-CoV-2. Combined the high safety and convenience, VEEV-SARS-CoV-2-S-eGFP may provide a potent platform for SARS-CoV-2 diagnosis, antiviral screening and the syncytium study.

## Materials and methods

**Cells and antibodies.** BHK-21 and Vero-E6 cells were grown in Dulbecco’s modified Eagle’s medium (DMEM) (Gibco) containing 10% heat-inactivated fetal bovine serum (FBS) (Gibco), 100 units/ml of penicillin, 100 µg/ml of streptomycin in 5% CO_2_ at 37°C. Anti-SARS-CoV-2 RBD monoclonal antibody was kindly provided by Prof. Bing Yan (Wuhan Institute of Virology, Chinese Academy of Sciences, Wuhan, China). The specific anti-hACE2 antibody was kindly provided by Prof. Chun-He Wang (Shanghai Institute of Materia Medica, Chinese Academy of Sciences, Shanghai, China). Texas Red-conjugated goat anti-mouse IgG secondary antibodies were purchased from Proteintech (China).

**Construction and passage of the chimaeric VEEV-SARS-CoV-2-S and VEEV-SARS-CoV-2-S-eGFP.** VEEV-SARS-CoV-2-S plasmid was constructed using the standard recombinant DNA technique. For VEEV replicon (pACYC-VEEV-Rep), the infectious clone of the VEEV attenuated vaccine strain TC83 [20] (GenBank No: L01443.1), designated as pACYC-VEEV-TC83, which carries a *AscI* restriction site downstream of the subgenomic (SG) promoter and a *PacI* restriction site upstream of the 3ʹUTR, was used for the construction capable of expressing SARS-CoV-2 S protein. The S glycoprotein sequence of SARS-CoV-2 (WIV04, GenBank No: MN996528.1) with a 21-amino-acid deletion at its C-terminus which has been shown to likely facilitate more efficient incorporation of the SARS-CoV-2 S protein into the VSV particles [21–23] was in vitro synthesized from Shenggong, China, and inserted into pACYC-VEEV-TC83 at *AscI* and *PacI* restriction sites to replace the structural genes of VEEV, generating the VEEV-SARS-CoV-2-S vector. The resulting plasmid VEEV-SARS-CoV-2-S was verified by DNA sequencing. To express the enhanced green fluorescent protein (eGFP) simultaneously, a second SG promoter was inserted downstream the original SG promoter into the VEEV replicon vector, generating the VEEV-SARS-CoV-2-S-eGFP replicon. As a control, the eGFP only VEEV replicon (VEEV-eGFP) was also constructed like VEEV-SARS-CoV-2-S. The cDNA plasmids were subjected to sequential *NotI* linearization and *in vitro* transcription using a mMESSENGER mMACHINE T7 Kit (Invitrogen) following the manufacturer’s instructions.

In our previous study, we found that the CHIKV genome with a complete capsid deletion (ΔC-CHIKV) could produce infectious particles that were genetically stable, highly attenuated, immunogenic, and able to confer complete protection against lethal CHIKV challenge [24]. Also, VEEV-RABV-G, constructed by the same strategy as VEEV-SARS-CoV-2-S, was proved to be safe and effective in mouse model, and can be a promising RABV vaccine candidate for human and domestic animal use [19]. Based on these findings, we performed all the following experiments in a BSL-2 laboratory.

For transfection of Vero-E6 cells, 8 × 10^6^ cells in 0.8 ml cold Ingenio electroporation solution (Mirus) were electroporated with 10 μg of RNA in a 4-mm cuvette at settings of 270 V and 950 μF with single electrical pulse using a GenePulser Xcell system (BIO-RAD). The electroporated cells were seeded into a T-75 flask and incubated at 37°C in 5% CO_2_ for daily observation.

Serial cell passages and supernatant passages were performed as the following procedure. The VEEV-SARS-CoV-2-S or VEEV-SARS-CoV-2-S-eGFP genomic RNA-transfected Vero-E6 cells or the culture medium collected at 72 h post-transfection (hpt) were designated as P0 and used for the following passages. For cell passages, the P0 replicating cells were then mixed with naïve Vero-E6 cells at a ratio of 1:10. When 70–80% cells displayed cell–cell membrane fusion, cells were subcultured into a new round of passage (P1-P5 for VEEV-SARS-CoV-2-S; P1-P20 for VEEV-SARS-CoV-2-S-eGFP). For supernatant passages, naïve Vero-E6 cells growing in 35 mm cell culture dishes were infected with 500 μl of culture medium from the previous passage and incubated for 3–4 days. The same passage procedure was also performed for VEEV-eGFP.

**Indirect immunofluorescence assay (IFA).** The VEEV-SARS-CoV-2-S genomic RNA electroporated Vero-E6 cells, the blind cell passaged or supernatant passaged Vero-E6 cells were seeded into 35 mm cell culture dishes containing coverslips (10 × 10 mm). At the indicated time points, the coverslips containing transfected or passaged cells were washed three times with PBS, followed by fixation with cold 5% acetone in methanol for 15 min at room temperature. Then, the fixed cells were stained with RBD antibody for 1 h at room temperature, washed three times with PBS, and incubated with Texas Red-conjugated goat anti-mouse IgG polyclonal secondary antibody. After three washes with PBS, the coverslips were mounted on glass slides with 90% glycerol. Images were captured under a fluorescence microscope (Nikon Eclipse TE2000).

**Inhibition assay of NH_4_Cl and antiviral agents.** VEEV-SARS-CoV-2-S-eGFP cell monolayers were incubated with 20–60 mM of NH_4_Cl, different concentrations (1 μM/10 μM) of antiviral medicines including cepharanthine, remdesivir, berberine hydrochloride, fangchinoline, isoliensinine, liensinine and tetrandrine, or 10 μg/ml of hACE2 antibody. To rule out pleiotropic effects, the VEEV-eGFP replicon (an eGFP only VEEV replicon) transfected Vero-E6 cells were treated with the same concentration of NH_4_Cl, cepharanthine and remdesivir. The eGFP expression and syncytium formation were observed under a ﬂuorescent microscope at 18 h post treatment. The aggregate fluorescence intensity in 169 fields of view (cover almost the whole well) per well in a 12-well plate with or without hACE2 Ab was read by a PerkinElmer high content screening system.

**Measurement of VEEV-SARS-CoV-2-S-eGFP cell–cell fusion in BHK-21 cells.** The naïve BHK-21 cells were transfected with pCAGGS-hACE2 plasmid using the FuGENE® HD Transfection Reagent (Promega) one day before the transfection of VEEV-SARS-CoV-2-S-eGFP or VEEV-eGFP RNA with DMRIE-C (Invitrogen) reagent. The expression of GFP was scored under a ﬂuorescent microscope at 48 and 72 h post RNA transfection and the integrated fluorescence intensity was quantified by ImageJ software.

**VEEV-SARS-CoV-2-S-eGFP neutralization assay.** Serial tenfold dilutions of heat-inactivated (56°C for 30 min) serum samples (starting at a 1:30 dilution) against SARS-CoV-2 were incubated with VEEV-SARS-CoV-2-S-eGFP replicating cells for 18 h at 37°C. The neutralizing activities were characterized by the decay of GFP ﬂuorescence signals relative to the control.

**Statistical analysis**. The unpaired *t*-test or two-way ANOVA was used to determine whether there were significant differences (*p* < 0.05) in all experiments. The statistical analyses were performed using non-parametric test in GraphPad Prism software 5.0.

## Results

### Generation of a hybrid replicon VEEV-SARS-CoV-2-S with a steady capability to induce cell–cell fusion and syncytium formation

**Figure 1.**
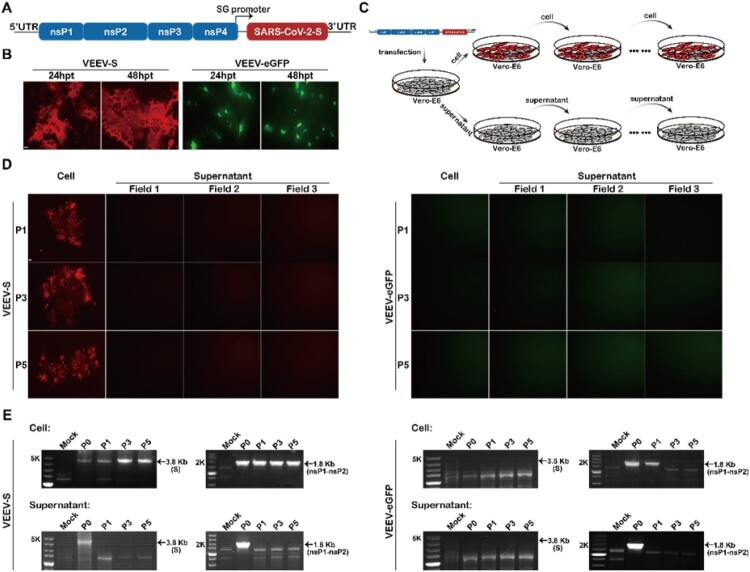
Generation and characterization of a hybrid replicon VEEV-SARS-CoV-2-S. (A) Schematic representation of the genomic organization of VEEV-SARS-CoV-2-S in which the structural protein sequences were replaced by the gene of SARS-CoV-2 S protein. (B) IFA analysis of the expression of S protein in VEEV-SARS-CoV-2-S RNA-transfected Vero-E6 cells using RBD monoclonal antibody at the indicated times post-transfection. The expression of GFP in the control replicon RNA (VEEV-eGFP)-transfected Vero-E6 cells was also measured. The length of the scale bar (displayed in a white line segment) represents 20 μm. (C) Schematic illustration of VEEV-SARS-CoV-2-S cell passage and supernatant passage. The VEEV-eGFP replicon was used as a negative control that underwent the same procedure. (D) IFA analysis of the expression of S protein in VEEV-SARS-CoV-2-S replicating Vero-E6 cells at different passages using RBD monoclonal antibody. The expression of GFP in VEEV-eGFP passaged cells was used as a control. The length of the scale bar (displayed in a white line segment) represents 50 μm. Two independent experiments were performed and the cells analyzed from one or three fields of view in one coverslip of one experiment are presented. (E) RT-PCR analysis of the stability of S and nsP1-nsP2 genes during passage. The same analysis for VEEV-eGFP was used as a control.

In previous studies, it has been demonstrated the feasibility of PRPs or iMVs strategy based on a modified alphavirus replicon system for antigen expression, in which the alphavirus envelope protein, or the heterogenous viral structural protein like rabies virus glycoprotein (RABV-G) was used as the only structural protein to assemble alphavirus replicon RNA into self-replicating PRPs or iMVs [19,24,25]. Using the same strategy, here we constructed a VEEV replicon expressing SARS-CoV-2 S protein (VEEV-SARS-CoV-2-S). As shown in Figure 1(A), the gene of S protein was inserted downstream of the 26S subgenomic (SG) promoter in place of the genes of VEEV structural proteins. After electroporation of the chimaeric VEEV-SARS-CoV-2-S replicon RNA into Vero-E6 cells, the replication capability of VEEV-SARS-CoV-2-S RNA and the expression of S protein were measured at 24 and 48 h post-transfection (hpt) by indirect immunofluorescence assay (IFA) using a monoclonal antibody against the RBD domain of S protein. VEEV-SARS-CoV-2-S RNA produced increasing numbers of IFA-positive cells featured by syncytial fusion in contrast to the scattered eGFP fluorescence in VEEV-eGFP RNA transfected cells (Figure 1(B)), indicating the propagation of VEEV-SARS-CoV-2-S in transfected Vero-E6 cells. In addition, the occurrence of syncytia in VEEV-SARS-CoV-2-S transfected cells also showed that the VEEV-SARS-CoV-2-S RNA has the same ability to induce syncytium formation in Vero-E6 cells as S-expressing plasmids or other replication-competent vesicular stomatitis virus (VSV)-based vectors bearing S gene [21,22,26,27]. However, no free infectious iMVs were released even after the supernatants harvested from the transfected cells had undergone 5 rounds of passage in Vero-E6 cells (Figure 1(D), left panel). In contrast, VEEV-SARS-CoV-2-S always retained the capability to induce cell–cell fusion and syncytium formation as the percentage of syncytia-like IFA-positive cells from one field of view was about 30–40% in naïve Vero-E6 cells inoculated with each passage of cells (Figure 1(D), left panel). RT–PCR assay was carried out using two primer pairs amplifying the complete S coding sequence and partial nonstructural protein nsP1-nsP2 sequence, respectively. Consistently, the 3.8-kb RT–PCR products for S and 1.8-kb RT–PCR products for nsP1-nsP2 were only detectable in cell passaged VEEV-SARS-CoV-2-S samples (Figure 1(E)). Additionally, the same passage (Figure 1(C)) was performed on a negative control, VEEV-eGFP replicon. As expected, except for gradually decreased nsP1-nsP2 RT–PCR product yields in cell passaged samples (P0-P3), neither GFP fluorescence (Figure 1(D), right panel) nor RT–PCR products (Figure 1(E), right panel) were detected in cell or supernatant passaged VEEV-eGFP samples. Though still unknown about the underlying mechanism, it is definitely different from either previous VSV-S system in which both clear cytopathic effects (CPE) and syncytia were visible [21,22,28]. Overall, our results suggest that VEEV-SARS-CoV-2-S system is featured with a steady capability to induce cell–cell fusion and syncytium formation.

### Generation of VEEV-SARS-CoV-2-S-eGFP reporter system facilitating cell–cell fusion monitor

**Figure 2.**
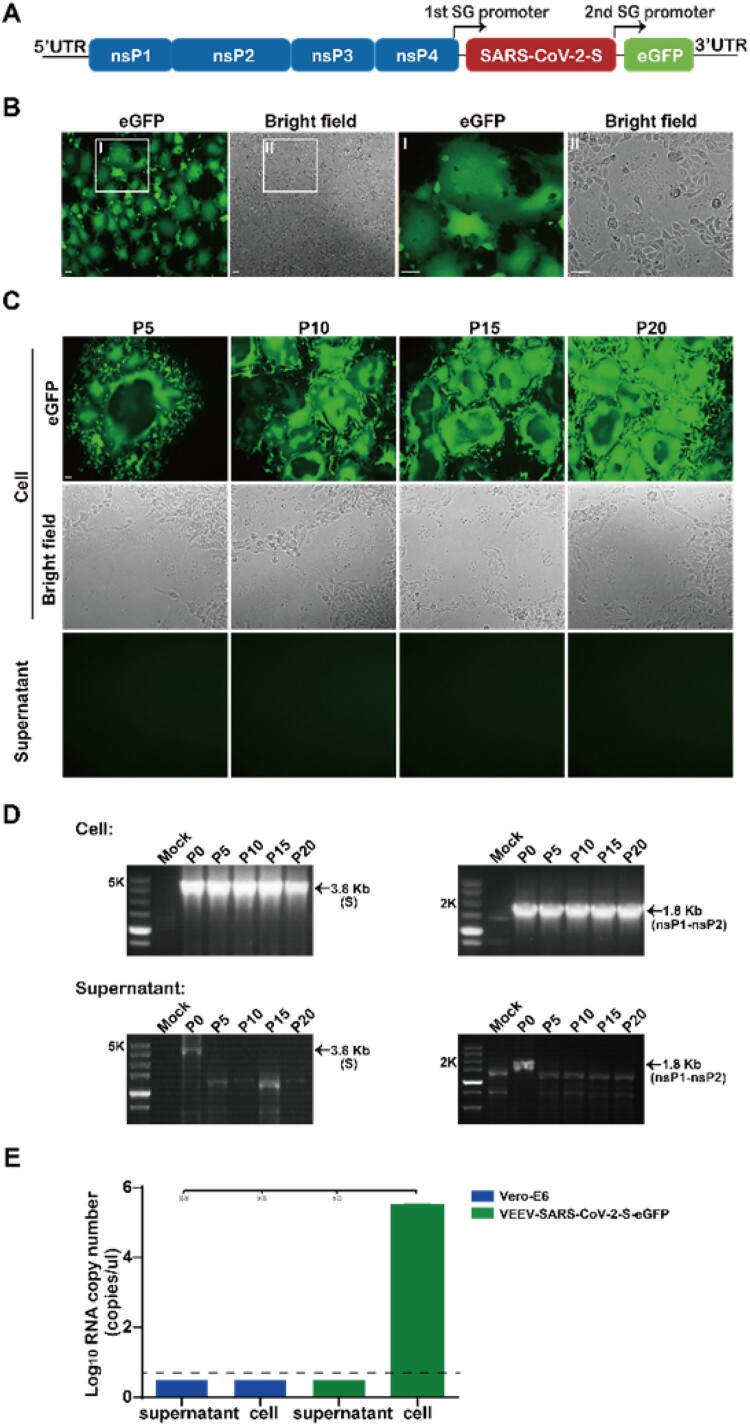
Generation and characterization of VEEV-SARS-CoV-2-S-eGFP reporter system. (A) Schematic diagram of the VEEV-SARS-CoV-2-S-eGFP genome. The expression of an enhanced green fluorescent protein (eGFP) was driven by a second SG promoter in the VEEV-SARS-CoV-2-S genome. (B) Detection of GFP expression and syncytium formation under a ﬂuorescent microscope in VEEV-SARS-CoV-2-S-eGFP RNA transfected Vero-E6 cells at 48 hpt. The magnified fields of view (I and II) were shown at the right two panels. The length of the scale bar (displayed in a white line segment) represents 50 μm. (C) Detection of GFP expression and syncytium formation in VEEV-SARS-CoV-2-S-eGFP replicating cells at each passage under a ﬂuorescent microscope. The length of the scale bar (displayed in a white line segment) represents 50 μm. (D) RT-PCR analysis of the stability of S and nsP1-nsP2 genes during passage. (E) Viral quantification of the supernatant and cell RNA extracted from the P20 VEEV-SARS-CoV-2-S-eGFP. Viral RNA loads in P20 VEEV-SARS-CoV-2-S-eGFP supernatants and cells were determined by a real-time reverse transcription (RT)-PCR assay. The Vero-E6 supernatants and cells were used as a negative control. Two independent experiments were performed in duplicate. Data represent the mean ± standard deviation of duplicate measurements in a representative experiment. Statistical analysis was performed with unpaired *t*-test and the asterisks denote statistical differences between the indicated groups. **p < 0.01.

To facilitate cell–cell fusion monitor, an eGFP reporter gene was inserted under a second SG promoter of VEEV-SARS-CoV-2-S, generating VEEV-SARS-CoV-2-S-eGFP reporter system (Figure 2(A)). Following transfection of *in vitro* transcribed VEEV-SARS-CoV-2-S-eGFP replicon RNA into Vero-E6 cells, the replication capability of VEEV-SARS-CoV-2-S-eGFP RNA was measured by directly monitoring the expression of GFP under a fluorescent microscope. Similarly, robust GFP positive syncytia were also observed within VEEV-SARS-CoV-2-S-eGFP transfected cells at 48 hpt (Figure 2(B)). To further investigate the stability of VEEV-SARS-CoV-2-S-eGFP replicon, the transfected cells or supernatants were passaged for 20 rounds using the same method as that for VEEV-SARS-CoV-2-S (Figure 1(C)) and the GFP expression was monitored at each passage. It showed that VEEV-SARS-CoV-2-S-eGFP also propagated through cell–cell fusion, causing visible GFP positive syncytia (Figure 2(C), upper panel) together with the apparent RT–PCR products of S and nsP1-nsP2 genes from each cell passaged sample (Figure 2(D), upper panel). Neither GFP positive cells nor RT–PCR products were detected in supernatant passaged samples (Figure 2(C,D), lower panel). To further confirm there were no iMVs releasing, we extracted the virion RNAs from the supernatants and cells of the P20 VEEV-SARS-CoV-2-S-eGFP and detected the viral RNAs with a real-time reverse transcription (RT)-PCR assay. As shown in Figure 2(E), like those extracted from naïve Vero-E6 cell lysates and supernatants, the viral RNA levels extracted from VEEV-SARS-CoV-2-S-eGFP P20 supernatants were below the limit of detection, in contrast to the considerably high level of viral RNA copy numbers (>10^5^ copies/μl) in VEEV-SARS-CoV-2-S-eGFP P20 cell lysates. Together, VEEV-SARS-CoV-2-S-eGFP reporter system has the same capability to form syncytia as VEEV-SARS-CoV-2-S vector characterized by the genetical stability without iMVs production and direct eGFP detection, which could be used to surrogate for cell–cell transmission and syncytium formation.

### Cell–cell fusion and syncytium formation induced by VEEV-SARS-CoV-2-S-eGFP is S-and ACE2-dependent

It has been demonstrated that the formation of syncytia during SARS-CoV-2 infection requires both expressions of viral S protein and host ACE2 protein in donor and acceptor cells, respectively [29–31]. To further confirm whether VEEV-SARS-CoV-2-S-eGFP induced cell–cell fusion and syncytium formation can simulate the events induced by authentic SARS-CoV-2 infection, we performed the following assays.

**Figure 3.**
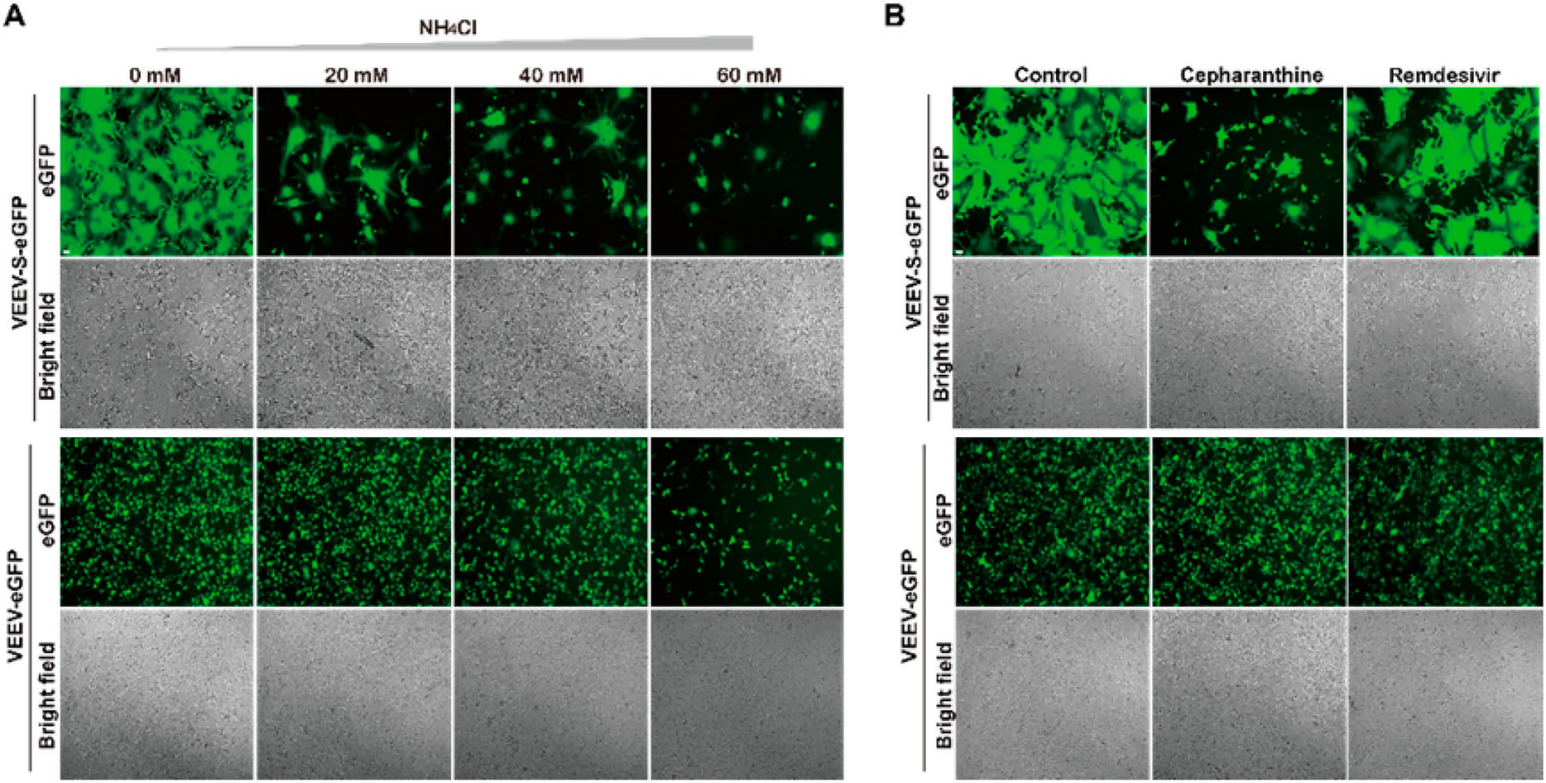
Cell-cell fusion and syncytium formation induced by VEEV-SARS-CoV-2-S-eGFP require S protein in an acidic environment. (A) Detection of GFP expression and syncytium formation in VEEV-SARS-CoV-2-S-eGFP cells in the presence of NH_4_Cl. 1 × 10^5^ VEEV-SARS-CoV-2-S-eGFP cells and 2 × 10^5^ naïve Vero-E6 cells were co-seeded per well in a 12-well plate in the presence of different concentrations of NH_4_Cl. The VEEV-eGFP replicon transfected Vero-E6 cells with the same treatment were used to rule out pleiotropic effects. The GFP expression and syncytium formation were detected under a ﬂuorescent microscope at 18 h post incubation. The length of the scale bar (displayed in a white line segment) represents 50 μm. (B) Detection of GFP expression and syncytium formation in VEEV-SARS-CoV-2-S-eGFP cells in the presence of different compounds. 1 × 10^5^ VEEV-SARS-CoV-2-S-eGFP cells and 2 × 10^5^ naïve Vero-E6 cells were co-seeded per well in a 12-well plate in the presence of 10 μM of cepharanthine and remdesivir, respectively. The VEEV-eGFP replicon transfected Vero-E6 cells with the same treatment were used to rule out pleiotropic effects. The GFP expression and syncytium formation were detected under a ﬂuorescent microscope at 18 h post incubation. The cells without treatment were used as a negative control. The length of the scale bar (displayed in a white line segment) represents 50 μm. Two independent experiments were performed, and one representative experiment is presented.

Firstly, given that the S protein-mediated entry requires the proteolytic activity of acidic-dependent endosomal cysteine cathepsins [31,32], an endosomal acidification inhibitor, NH_4_Cl, was used to test its effect on syncytium formation induced by VEEV-SARS-CoV-2-S-eGFP. As shown in Figure 3, treatment with NH_4_Cl inhibited efficiently the formation of syncytia in a dose-dependent manner, characterized by decreasing numbers of syncytia-like GFP positive cells (Figure 3(A), upper panel) and the same inhibitory effects were also observed when treated with a membrane fusion inhibitor, cepharanthine [33] (Figure 3(B), upper panel). In contrast, no GFP signal decrease was observed in 20 and 40 mM NH_4_Cl- and cepharanthine- treated VEEV-eGFP replicon transfected group (Figure 3(A,B), lower panel). As expected, remdesivir that targets viral nsP12 protein (viral RNA-dependent RNA polymerase, RdRP) [34] had little effect on syncytium formation and GFP signals (Figure 3(B), right panel). These data demonstrate VEEV-SARS-CoV-2-S-eGFP induced syncytium formation is S-dependent.

**Figure 4.**
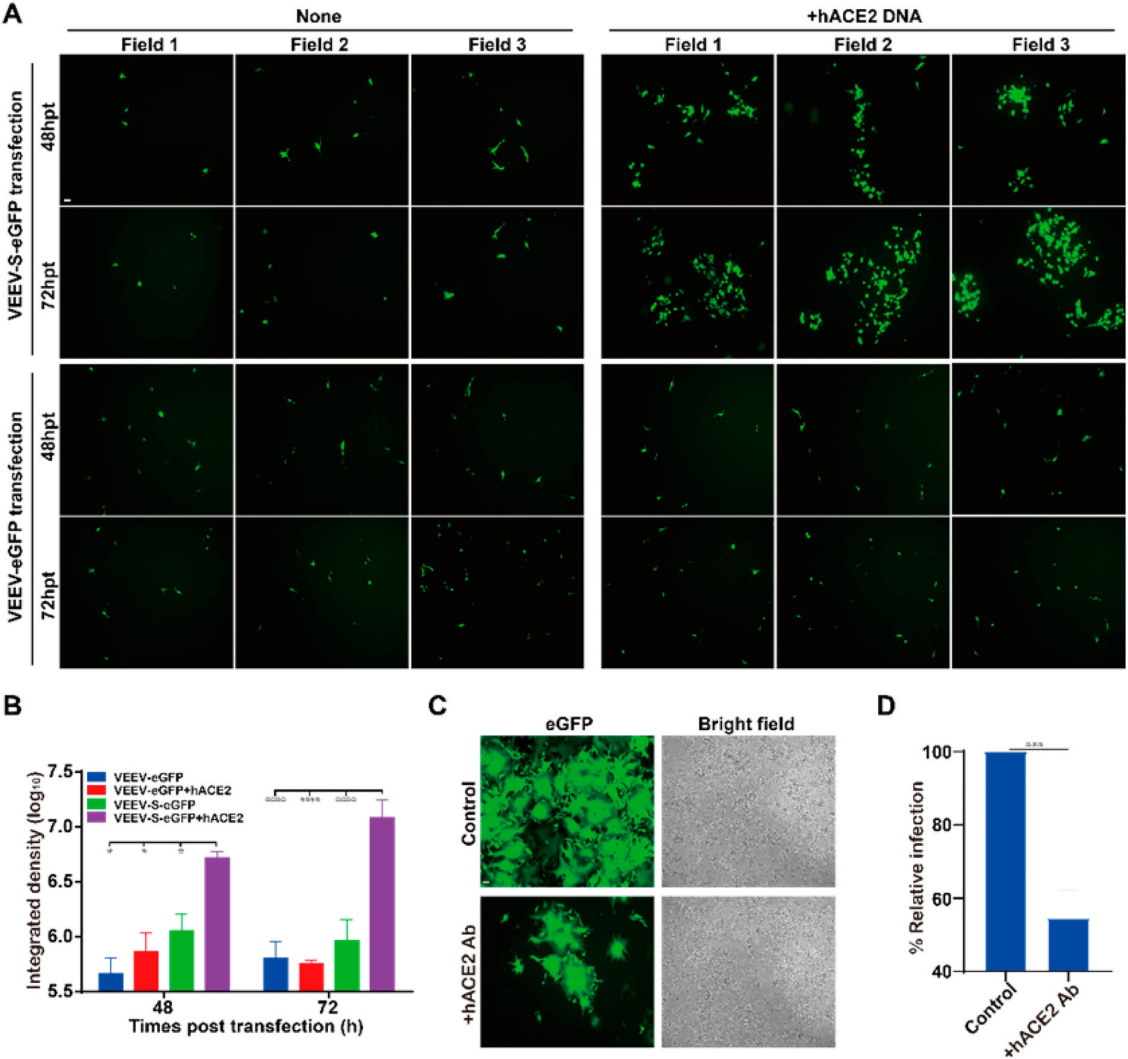
Cell-cell fusion and syncytium formation induced by VEEV-SARS-CoV-2-S-eGFP is hACE2-dependent. (A) GFP detection of the VEEV-SARS-CoV-2-S-eGFP transfected naïve BHK-21 cells or BHK-21 cells pre-transfected with hACE2 cDNA. BHK-21 cells were transfected with pCAGGS-hACE2 plasmid by the FuGENE® HD Transfection Reagent one day before the transfection of VEEV-SARS-CoV-2-S-eGFP RNA. The cell-cell fusion was presented by the expression of GFP at 48 and 72 hpt under a ﬂuorescent microscope. The VEEV-eGFP transfected naïve BHK-21 cells or BHK-21 cells pre-transfected with hACE2 cDNA were used as a negative control. The length of the scale bar (displayed in a white line segment) represents 50 μm. Two independent experiments were performed and 3 fields in one representative experiment are presented. (B) Quantification of GFP fluorescence intensity. The integrated fluorescence intensity in panel A was quantified by ImageJ software. Data represent the mean ± standard deviation of 3 fields in the representative experiment. Statistical analysis was performed with two-way ANOVA and the asterisks denote statistical differences between the indicated groups. *p < 0.05; ****p < 0.0001. (C) Detection of GFP expression and syncytium formation in VEEV-SARS-CoV-2-S-eGFP cells in the presence of anti-human ACE2 antibody (hACE2 Ab). 1 × 10^5^ VEEV-SARS-CoV-2-S-eGFP cells and 2 × 10^5^ naïve Vero-E6 cells were co-seeded per well in a 12-well plate in the presence of 10 μg/ml of hACE2 Ab, and imaged by a ﬂuorescent microscope at 18 h post incubation. The cells without treatment were used as a control. The length of the scale bar (displayed in a white line segment) represents 50 μm. Two independent experiments were performed in duplicate, and one representative experiment is presented. (D) Calculation of GFP fluorescence intensity. At 18 h post treatment, the aggregate fluorescence intensity in 169 fields of view (cover almost the whole well) per well in a 12-well plate with or without hACE2 Ab was read by a PerkinElmer high content screening system and represented by the relative infection ratio (defining no-antibody treatment as 100%). Data represent the mean ± standard deviation of duplicate measurements in a representative experiment. Statistical analysis was performed with unpaired *t*-test and the asterisks denote statistical differences between the indicated groups. ***p < 0.001.

Secondly, the receptor-dependent syncytium formation of VEEV-SARS-CoV-2-S-eGFP was determined through two methods: one is hACE2 expression method, and the other is antibody inhibition method. For the hACE2 expression method, a SARS-CoV-2 non-susceptible BHK-21 cell line expressing low levels of ACE2 mRNA [21,31] was used instead of the susceptible Vero-E6 cell line. We transfected VEEV-SARS-CoV-2-S-eGFP replicon RNA into BHK-21 cells, and only scattered GFP positive cells were observed in transfected BHK-21 cells at each time point post transfection (Figure 4(A), upper left panel; Figure 4(B)). In contrast, when BHK-21 cells were transfected with hACE2 expression plasmid prior to transfection with VEEV-SARS-CoV-2-S-eGFP replicon RNA, a time-dependent increase in GFP positive cell clusters was detected (Figure 4(A), upper right panel; Figure 4(B)). As a negative control, we also transfected VEEV-eGFP replicon RNA into naïve BHK-21 cells or BHK-21 cells pre-transfected with hACE2 expression plasmid, and only scattered GFP positive cells were detected at each time point post transfection (Figure 4(A), lower panel; Figure 4(B)). For the antibody inhibition method, a neutralizing antibody against hACE2, 3E8 [35] was incubated with VEEV-SARS-CoV-2-S-eGFP replicating cells for 18 h. As shown in Figure 4(C,D), the addition of 3E8 greatly reduced syncytium formation compared with the antibody-absent control. Collectively, these results provide strong evidence that VEEV-SARS-CoV-2-S-eGFP induced syncytium formation can mimic what the authentic SARS-CoV-2 does in a S- and receptor-dependent manner.

### Validation of VEEV-SARS-CoV-2-S-eGFP system for assessing the anti-syncytium-formation activity of antiviral agents

**Figure 5.**
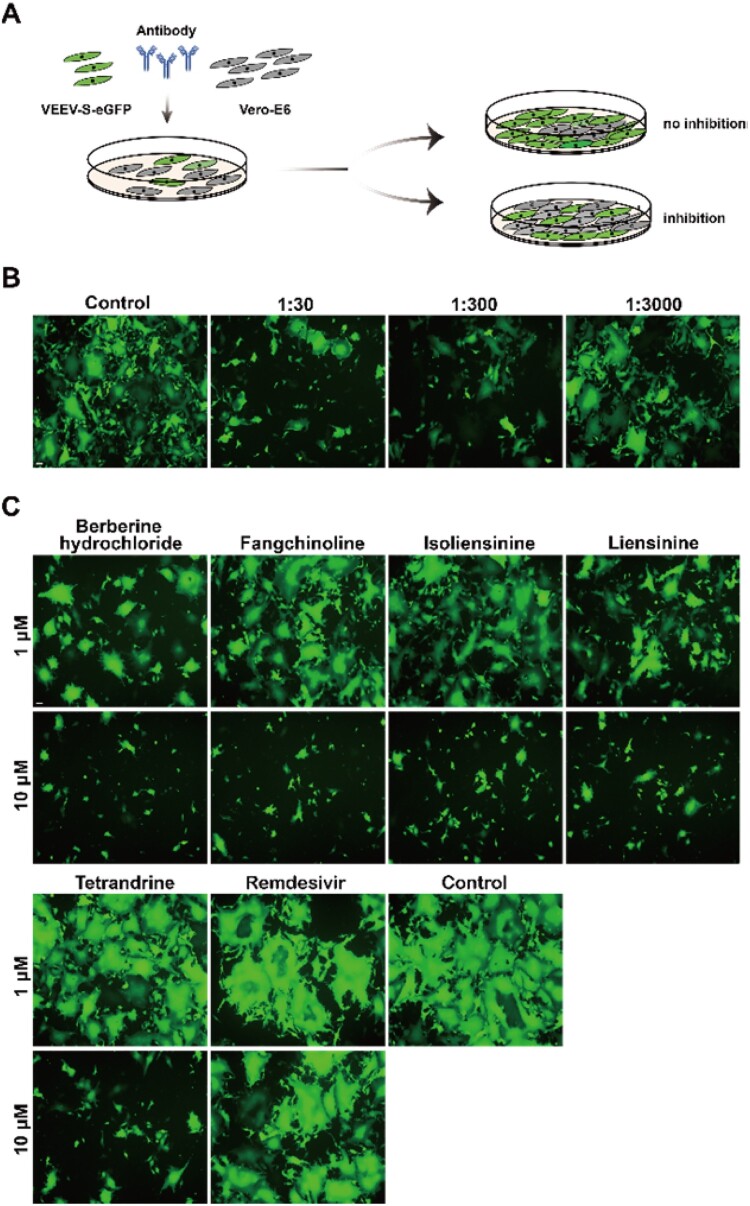
Validation of VEEV-SARS-CoV-2-S-eGFP system for assessing the anti-syncytium-formation activity of antiviral agents. (A) Schematic diagram of the neutralization assay. 1 × 10^5^ VEEV-SARS-CoV-2-S-eGFP cells and 2 × 10^5^ naïve Vero-E6 cells were co-seeded per well in a 12-well plate in the presence of mouse antisera, the GFP expression was detected under a fluorescent microscope at 18 h post incubation. Serum from mock-infected mice was used as a control. (B) Measurement of antiserum neutralizing activities. The length of the scale bar (displayed in a white line segment) represents 50 μm. (C) Measurement of anti-syncytium-formation activity of entry inhibitors. 1 × 10^5^ VEEV-SARS-CoV-2-S-eGFP cells and 2 × 10^5^ naïve Vero-E6 cells were co-seeded per well in a 12-well plate in the presence of 1 μM or 10 μM of inhibitors, and imaged by a ﬂuorescent microscope at 18 h post incubation. The cells without treatment were used as a control. The length of the scale bar (displayed in a white line segment) represents 50 μm. Two independent experiments were performed in duplicate, and one representative experiment is presented.

Then we examined the feasibility of this system for the evaluation of anti-syncytium-formation agents. Firstly, we tested the antiviral activity of neutralizing antibody using this system by incubating the VEEV-SARS-CoV-2-S-eGFP replicating cells with different dilutions of mouse antisera against SARS-CoV-2 (Figure 5(A)). As shown in Figure 5(B), a dose-dependent inhibition of GFP-positive syncytia was observed upon incubation with antiserum, indicating that our system could be used for neutralizing assay. Secondly, multiple antiviral agents targeting viral entry demonstrated in our study (unpublished data) or in previous reports [36–39] were used to test the feasibility of this system. As expected, all entry inhibitors blocked syncytium formation in a dose-dependent manner, characterized by differential decay of GFP signals in VEEV-SARS-CoV-2-S-eGFP replicating cells, while the negative remdesivir treatment group had little effect on the formation of syncytia (Figure 5(C)). Taken together, these data demonstrate that VEEV-SARS-CoV-2-S-eGFP replicating cells provide a convenient and reliable platform for the rapid identification of inhibitors blocking S-mediated cell–cell membrane fusion and syncytium formation.

## Discussion

With the increasing understanding of the relationship between the S-driven syncytium formation and severe respiratory dysfunction and the high rate of virus spread [7,8], it becomes urgent to develop the platform for the study of the underlying mechanism and the screening for drugs blocking syncytia [9]. In this study, we established a new platform based on VEEV-SARS-CoV-2-S-eGFP replicating cells for screening syncytia-inhibitors that is proved to be reliable, efficient, and convenient.

Because it has been demonstrated that the S protein itself can trigger the formation of syncytia without any other viral proteins, the mammalian expression system has become the most prevalent method in syncytium study due to its safety handling [23,27,40], but it requires repeated transfection and therefore is tedious and costly. Instead, different versions of replication-competent VSV/S chimaera were also developed for studies of S-mediated cell entry and its inhibition. Transfection of VSV/S genomic RNA often generated a combination of cytopathic effects (CPE) and syncytia phenotypes. Following serial passages, the infectious VSV/S virions were easily selected and propagated, eventually establishing infectious chimaeric virions expressing S protein system [21,22,28]. Here the strategy we used to construct VEEV-SARS-CoV-2-S-eGFP vector was based on a modified alphavirus replicon system that has been utilized for the development of live attenuated vaccine candidates of Rabies virus (VEEV-RABV-G) [19] and Chikungunya virus (ΔC-CHIKV) [24]. Despite using the same strategy, VEEV-SARS-CoV-2-S-eGFP RNA did not generate iMVs, as previous vectors did, instead, it propagated through cell-to-cell transmission pathway, forming GFP positive syncytia in S- and receptor-dependent manner (Figure 3 and Figure 4). Such syncytium-forming ability in Vero-E6 cells was able to be retained for at least 20 passages without free iMVs releasing (Figure 2). Moreover, a significant dose-dependent decay in GFP signals was observed in VEEV-SARS-CoV-2-S-eGFP replicating cells upon treatment with SARS-CoV-2 antiserum or antiviral drugs targeting virus entry, providing strong evidence that VEEV-SARS-CoV-2-S-eGFP system is highly sensitive to characterize the anti-syncytium-formation activity of antiviral agents (Figure 5).

Although it is unknown about the reason causing such differences between VEEV-SARS-CoV-2-S-eGFP and the other previous VEEV-based expression systems or VSV/S chimaera, its unique syncytium-forming ability offers a more focused platform for syncytia study which requires a shorter time of incubation and allows the target of any inhibitor to be easily identified. In comparison with the mammalian system, its replication-competence greatly simplifies the experimental procedure by just cell passaging instead of labour-intensive transfection. Furthermore, compared to VSV/S chimaeric system that is involved in the manipulation of infectious chimaeric viruses, this VEEV-SARS-CoV-2-S-eGFP system is primarily cell-based without iMVs production even after 20 rounds of serial passages in Vero-E6 cells. Such features undoubtedly further increase manipulation safety, allowing related studies to be conducted in BSL-2 laboratories and thus clearing a big hurdle for most SARS-CoV-2 studies requiring high biosafety level BSL-3 facilities.

In conclusion, we generated a new recombinant replication-competent VEEV-SARS-CoV-2-S-eGFP vector expressing the SARS-CoV-2 S protein. It could induce cell–cell fusion and syncytium formation in susceptible cells in the same way as the authentic SARS-CoV-2 does, providing a safe, rapid and targeted platform for the study of the underlying mechanism and the screening for drugs blocking syncytia.

## Data Availability

All data to understand and assess the conclusions of this study are available in this published article. The raw data that support the findings of this study are available from the corresponding author upon reasonable request.
